# Performance of Malnutrition Screening Tools on People With Chronic Diseases: A Bivariate Meta-Analysis

**DOI:** 10.1097/jnr.0000000000000728

**Published:** 2026-02-04

**Authors:** Hidayat Arifin, Ruey Chen, Chien-Mei Sung, Kai-Jo Chiang, Kuei-Ru Chou

**Affiliations:** 1 School of Nursing, College of Nursing, Taipei Medical University, Taiwan; 2 Department of Medical-Surgical, Emergency, Disaster, and Critical Nursing, Research Group in Medical-Surgical Nursing, Faculty of Nursing, Universitas Airlangga, Indonesia; 3 Department of Nursing, Taipei Medical University–Shuang Ho Hospital, Taiwan; 4 Post-Baccalaureate Program in Nursing, College of Nursing, Taipei Medical University, Taiwan; 5 College of Nursing, National Defense Medical University, Taipei, Taiwan; 6 Department of Nursing, Tri-Service General Hospital, Taiwan; 7 Research Center in Nursing Clinical Practice, Wan Fang Hospital, Taipei Medical University, Taiwan; 8 Psychiatric Research Center, Taipei Medical University Hospital, Taiwan; 9 Research Center for Neuroscience, Taipei Medical University, Taiwan

**Keywords:** malnutrition, Malnutrition Screening Tool, Malnutrition Universal Screening Tools, Nutritional Risk Screening 2002, chronic disease

## Abstract

**Background:**

Malnutrition significantly impacts mortality and morbidity in patients with chronic diseases. Accurate screening tools are necessary for the early identification and management of this condition. However, limited meta-analyses on screening tools designed to assess malnutrition in patients with chronic diseases exist.

**Purpose:**

This study was designed to evaluate the clinical efficacy of several screening tools widely used to detect malnutrition in patients with chronic diseases in clinical settings. Three tools were addressed in this meta-analysis, including the Malnutrition Screening Tool (MST), Malnutrition Universal Screening Tools (MUST), and Nutritional Risk Screening 2002 (NRS-2002).

**Methods:**

Seven electronic databases, including CINAHL-EBSCO, Cochrane, Embase, OVID-MEDLINE, PubMed, Scopus, and Web of Science, were searched systematically from their respective inception dates to October 29, 2023. Studies designed to evaluate the sensitivity and specificity of malnutrition using the Patient-Generated Subjective Global Assessment as the reference standard were included. Bivariate and random effects were used to summarize the following outcome variables: sensitivity, specificity, positive likelihood ratio, negative likelihood ratio, diagnostic odds ratio, and area under the curve. All of the statistical analyses were conducted using STATA software.

**Results:**

Twenty-six studies were included in the analysis. The sensitivity of MST in detecting malnutrition (.78, 95% confidence interval [CI] [.62, .88]) was higher than that of either MUST (.74, 95% CI [.68, .80]) or NRS-2002 (.67, 95% CI [.61, .71]). The respective specificities of NRS-2002, MST, and MUST were comparable (.88, 95% CI [.83, .92] vs. .82, 95% CI [.64, .90] vs. .80, 95% CI [.73, .89]). Similarly, in terms of malnutrition detection accuracy, the three tools had similar areas under curve (.82, 95% CI [.79, .85] vs. .87, 95% CI [.84, .90] vs. .79, 95% CI [.75, .82], respectively). However, Fagan nomograms showing positive and negative likelihood ratios of <10 and >0.1 indicate all three screening tools have a minimal effect rating with regard to detecting malnutrition.

**Conclusions:**

MST, MUST, and NRS-2002 all present a good level of accuracy in detecting malnutrition in patients with chronic diseases. Thus, it is recommended that nurses, physicians, dieticians, and other health care workers use these tools in daily practice. Further investigations are warranted to validate these findings.

## Introduction

Malnutrition refers to an insufficient or excessive intake of nutrients, an imbalance in crucial nutrients within the body, or impairment in the body’s utilization of nutrients in metabolic processes (World Health Organization, 2020). Globally, the prevalence of malnutrition with comorbidities ranges from 39% to 53% (Bian et al., 2023). Specifically, the prevalence of malnutrition in patients with chronic kidney disease is 42.7% (Rashid et al., 2021), 44% in those with cancer (Bian et al., 2023), 30% in those with chronic obstructive pulmonary disease (Deng et al., 2023), and 32.5% in those with dementia (Arifin et al., 2024). Chronic diseases are the primary cause of malnutrition among hospitalized patients (Doley, 2019), with malnutrition linked to elevated mortality rates and prolonged hospital stays (Vong et al., 2022). However, understanding and effectively assessing malnutrition in chronic patients remains insufficient. Accurately detecting malnutrition using suitable screening tools remains a challenge during hospitalization (Bellanti et al., 2020; van Dronkelaar et al., 2023). Therefore, the utilization of screening tools with high sensitivity and specificity is critically important in the early detection of malnutrition in patients with chronic diseases in clinical settings.

At present, no gold standard exists for screening nutritional status (Ruan et al., 2021; Serón-Arbeloa et al., 2022). The Patient-Generated Subjective Global Assessment (PG-SGA) is a multidisciplinary reference standard used in the malnutrition assessment of patients with oncology and other chronic diseases (Lang et al., 2022). Also, the Malnutrition Screening Tool (MST; Ferguson et al., 1999), Malnutrition Universal Screening Tools (MUST; Elia, 2003), and Nutritional Risk Screening 2002 (NRS-2002; Kondrup et al., 2003) are widely utilized as screening tools in many countries to evaluate the nutritional status of hospitalized patients and are the most-frequently validated tools (Totland et al., 2022). These three tools have demonstrated feasibility, contain a limited number of questions, are user-friendly, exhibit high validity and reliability (Bolayir et al., 2019; Di Bella et al., 2020), and have been translated into many different languages. Their widespread implementation and robust evidence base render them essential in clinical practice, particularly for patients with chronic diseases, and justify their selection over alternative screening tools in this meta-analysis.

Diagnostic accuracy is a critical concept in the performance evaluation of screening tools, particularly in clinical settings where timely and accurate identification of malnutrition is essential for effective management (de Lara et al., 2023). Understanding sensitivity and specificity is crucial for evaluating diagnostic test performance. These measures, along with predictive values and likelihood ratios, help clinicians and nurses make informed decisions on patient care. The findings of this meta-analysis are expected to reinforce and extend previous evidence by corroborating the diagnostic accuracy of these screening tools in the context of assessing malnutrition status. Nakyeyune et al. (2021) evaluated MST in relation to PG-SGA and SGA; Cortés-Aguilar et al. (2024) evaluated MUST, Mini Nutritional Assessment-Short Form (MNA-SF), and NRS-2002 in relation to the Subjective Global Assessment (SGA) and European Society for Clinical Nutrition and Metabolism, respectively; and Ruan et al. (2021) and (Nakyeyune et al., 2022) assessed the accuracy of screening tools against various reference standards. In this meta-analysis, the accuracy of MST, MUST, and NRS-2002 is concurrently compared, with PG-SGA used as the reference standard, to provide a more comprehensive understanding of their relative performance. By integrating prior study data, this study builds upon prior research to address a critical gap in terms of the comparative evaluation of these widely used tools.

Health care professionals, especially nurses, play a crucial role in assessing nutrition status using validated screening tools to ensure effective nutrition assessment is part of the admission process and to identify patients at risk of malnutrition (Van Den Berg et al., 2023). Nurses are at the forefront of patient care. In assessing the nutritional status of admitted patients, nurses use validated screening tools designed to quickly and accurately identify patients who may be at risk of malnutrition. These tools, as part of a structured approach to nutritional assessment, facilitate the timely identification of patients in need of further nutritional assessment or support, ensuring their health needs are addressed early on in their care (Gbareen et al., 2021). Given the importance of accuracy in the screening tools employed in clinical settings, this meta-analysis was conducted to pool and compare the sensitivity and specificity of MST, MUST, and NRS-2002 in comparison with PG-SGA and to assess the clinical efficacy of these three screening tools in detecting malnutrition in patients with chronic diseases.

## Methods

The guidelines and recommendations outlined in the Preferred Reporting Items for Systematic Review and Meta-Analysis of Diagnostic Test Accuracy Studies statement (McInnes et al., 2018) were referenced in conducting this meta-analysis. As all the data used were taken from previously published articles, ethical approval was not separately required to conduct this study. The protocol for this meta-analysis was registered with the International Prospective Register of Systematic Reviews (PROSPERO; CRD42023434592).

### Data Sources and Search Strategy

A comprehensive search was conducted in seven databases, including CINAHL-EBSCO, Cochrane, Embase, OVID-MEDLINE, PubMed, Scopus, and Web of Science, from their respective dates of inception to October 29, 2023. No restrictions were placed on language or publication year. The search utilized Boolean algebra combinations with the following terms and relevant keywords from Medical Subject Headings, Subject Headings (MeSH), and Emtrees: (malnutrition OR malnourishment* OR undernutrition OR “nutritional deficiency*” OR “deficiency disease*” OR “nutrition disorders”) AND (“Mini Nutritional Assessment-Short Form” OR “MNA-SF” OR “Nutritional Risk Screening 2002” OR “NRS-2002” OR “Malnutrition Universal Screening Tools” OR MUST OR “Malnutrition Screening Tool” OR MST) AND (“Patient-Generated Subjective Global Assessment” OR “PG-SGA”) AND (“diagnostic tests, routine” OR “diagnosis” OR “ROC curve” OR “sensitivity and specificity” OR validity OR reliability OR “data accuracy” OR psychometrics). The example of specific search terms used across the seven databases and their respective syntaxes are presented in Appendix A and B, Supplemental Digital Content, http://links.lww.com/JNR/A7. To identify additional relevant articles, a forward citation search was conducted to identify articles referenced in specific articles, and a backward citation search was performed to review the reference lists of previous systematic reviews, meta-analyses, and other studies. Google Scholar searches were also performed to identify other potentially eligible studies. The Mini Nutritional Assessment-Short Form was not included in this meta-analysis due to the limited number of related studies.

### Eligibility Criteria

In this study, studies designed to evaluate the MST, MUST, and/or NRS-2002 meeting the following inclusion criteria were included: (a) included patients with chronic diseases, (b) conducted in a clinical setting, (c) reported estimated diagnostic accuracy, and (d) utilized PG-SGA as a reference standard. In cases of incomplete information, the corresponding author was contacted via email for clarification. Otherwise qualified studies were excluded if they met any of the following criteria: (a) nonrelevant topics, (b) nonrelevant tools, (c) nonrelevant study design, (d) no information related to the 2×2 table (true positive, true negative, false positive, and false negative), (e) lacking an index or reference test, or (f) included insufficient data. Two researchers independently performed the study selection.

### Reference Standard

The PG-SGA, a modified version of the Subjective Global Assessment (SGA), includes additional questions about the presence of nutritional symptoms and recent weight loss (Ottery, 1996). PG-SGA includes items such as weight, intake, symptoms, activities, and functional characteristics that are generally filled in by the patient, while weight loss scores, disease state, metabolic demand, and nutritional physical examination results are filled in by health professionals such as physicians, nurses, or dieticians (Ottery, 1996). PG-SGA subscale results are interpreted based on numerical scores, which may be combined to provide a global rating of a patient’s nutritional status. Based on the PG-SGA global score, a patient may be categorized as well-nourished (PG-SGA A), moderately malnourished (PG-SGA B), or severely malnourished (PG-SGA C). Also, the PG-SGA global score indicates the level of intervention required, using the following categories: 0–1 (*low risk*), 2–3 (*moderate risk*), 4–8 (*high risk*), and 9 or higher (*very high risk*; Bauer et al., 2002).

### Index Tests for Malnutrition Screening

The index tests used for screening malnutrition in this study consist of three widely used instruments, namely MST, MUST, and NRS-2002. MST was initially developed by Ferguson et al. (1999) to screen for malnutrition in medical and surgical patients. It was designed to be easy to use, cost-effective, noninvasive, applicable in heterogeneous populations, and quick and convenient for nonprofessional staff and patient families to use. MST comprises four questions, with scores >2 indicating risk of malnutrition (Ferguson et al., 1999). MST has demonstrated moderate overall validity, good generalizability, moderate agreement, moderate reliability, and strong quality of evidence (Skipper et al., 2020). However, MST has weaknesses in terms of using subjective questions and unsuitability for use on maternity and psychiatric patients (Skipper et al., 2020; Appendix C, Supplemental Digital Content, http://links.lww.com/JNR/A7).

MUST, initially developed by the British Association for Parenteral and Enteral Nutrition (Elia, 2003), assesses body mass index (BMI), unintentional weight loss, and the impact of acute illness, considering periods of not consuming food for more than five days. MUST scores are categorized into *low-risk* (0), *medium-risk* (1), and *high-risk* (≥2) ranges. It is a widely used screening tool for hospitalized patients and is also recommended for use in community settings (Chávez-Tostado et al., 2020; Serón-Arbeloa et al., 2022). MUST exhibits excellent agreement, high overall validity and reliability, fair generalizability, and fair quality of evidence (Elia, 2003; Skipper et al., 2020; Appendix D, Supplemental Digital Content, http://links.lww.com/JNR/A7).

NRS-2002, developed by Kondrup et al. (2003) to quickly and easily identify patients with malnutrition who are most likely to require nutritional support, consists of a preliminary phase to identify BMI <20.5, weight loss in the last 3 months, decreased appetite in the last week, and the presence of a serious illness. When any of these conditions are found in the preliminary phase, the assessment proceeds to the screening phase, which evaluates weight loss, BMI, and decreased food intake, resulting in a score of 0–3. Based on the results, a disease severity examination is conducted that generates a score of 0–3. The total score, adjusted for age in patients above 70 years (+1), is derived from the nutritional and disease severity assessments, with total scores <3 indicating no risk of malnutrition and ≥3 indicating at risk that requires nutritional support (Kondrup et al., 2003). NRS-2002 is recommended for hospitalized and critically ill patients (Stello et al., 2023) and has shown high sensitivity and specificity, albeit with limited generalizability and a fair strength of evidence (Bolayir et al., 2019; Skipper et al., 2020; Appendix E, Supplemental Digital Content, http://links.lww.com/JNR/A7).

### Data Extraction

Data extraction in this study was concurrently and independently conducted by two researchers. Initial data screening was conducted based on title and abstract, with duplicate studies eliminated. Next, identified articles with irrelevant topics or irrelevant tools or that were nonresearch studies were excluded. The remaining articles were then subject to a full-text evaluation, with those lacking 2×2 table numbers or sufficient data excluded. Any disagreements were resolved through discussions involving three researchers. Following this process, the relevant data were extracted from the included studies, including study details, country, tools used, comparator tools, settings, diseases studied, total sample size, percentage of females, mean age, and mean BMI (Appendix F, Supplemental Digital Content, http://links.lww.com/JNR/A7).

### Risk of Bias Assessment

Two researchers assessed the methodological quality of the included studies independently using the revised Quality Assessment Tool for Diagnostic Accuracy Studies-2 (QUADAS-2; Whiting et al., 2011), which consists of 14 items in four domains: patient selection, index test, reference standard, and study flow and timing. Responses to each item are categorized as “yes,” “no,” or “unclear.” In instances of disagreement, resolutions were reached through discussions among the two researchers and resolved by the third reviewer when a consensus could not be achieved.

### Statistical Analysis

The statistical analyses employed in this study were conducted in several stages. First, Review Manager 5.3 (The Cochrane Co-operation, Oxford, UK) was used to calculate true positive, true negative, false positive, and false negative values in the included studies containing limited information data. Second, pooled estimates of sensitivity and specificity, along with 95% CIs, were calculated using bivariate random effect models. The analysis then proceeded using user-written commands such as midas, found in Stata Software, version 16.1 (StataCorp LP, College Station, TX, USA). Q statistics and *I* ^*2*^ were used to calculate the heterogeneity of sensitivity and specificity, with an *I* ^*2*^ value close to 0% indicating homogeneity, below 25% indicating low heterogeneity, 25%–50% indicating moderate heterogeneity, and over 50% indicating high heterogeneity among the included studies (Higgins et al., 2003). Although plans were made to use moderator analysis, including subgroup and meta-regression to assess covariates such as country, sample size, gender, and mean age, these were not implemented due to the limited data, which could impact the relevance of the results. In addition, to enhance robustness, publication bias was analyzed using Deeks’ funnel plot asymmetry test, with a *p*-value <.1 indicating potential publication bias (Deeks et al., 2005).

Third, in evaluating the diagnostic accuracy of tests, sensitivity and specificity are critical measures of performance. Sensitivity refers to the ability of a test to correctly identify individuals with the disease (true positive rate), with high sensitivity indicating the test is effective at detecting that condition. Conversely, specificity refers to the ability to correctly identify individuals without the disease (true negative rate), with high specificity indicating the test is effective at ruling out that condition (Polit & Beck, 2017). The diagnostic odds ratio (DOR) represents the ratio of the odds of positive results to negative results derived from an analysis. The DOR value is always >0, with higher values indicative of a more effective screening test (Glas et al., 2003). Fourth, the summary receiver operating characteristic curve was used in this study to facilitate direct comparison by describing the relationship between sensitivity and specificity, as well as the area under the curve (AUC; Takwoingi et al., 2015). In terms of AUC values, >.97 indicates excellent accuracy, while values .93–.96 indicate very good accuracy, .75-.92 indicate good accuracy, and .50–.75 indicate reasonable accuracy (Jones & Athanasiou, 2005). Subsequently, a sensitivity analysis was conducted to evaluate the robustness of the included studies. Moreover, Fagan’s nomogram was employed to calculate and determine post-test probabilities as well as to evaluate the effect size rating of screening tools in detecting malnutrition. Positive likelihood ratios (+/LR) > 10 and negative likelihood ratios (–/LR) < 0.1 were indicative of a substantial effect rating; +/LR > 10 and –/LR > 0.1 or +/LR < 10 and –/LR < 0.1 were indicative of a moderate effect rating; and +/LR < 10 and –/LR > 0.1 were indicative of a minimal effect rating (Rubinstein et al., 2018).

## Results

### Characteristics of the Included Studies

**Figure 1:**
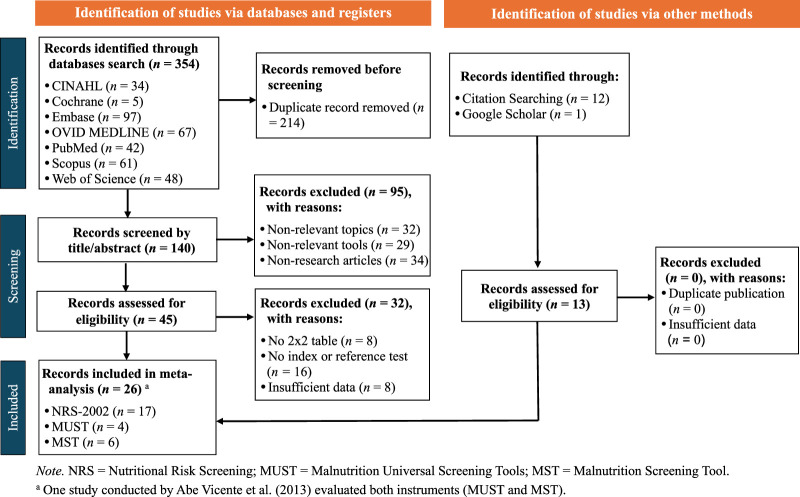
Flow Chart of Preferred Reporting Items for Systematic Reviews and Meta-Analyses

Details of the selection process for the included studies are presented in Preferred Reporting Items for Systematic Reviews and Meta-Analyses (Figure 1). Three hundred fifty-four articles were obtained in the initial search of the seven databases, with 214 articles excluded as duplicates. Of the 140 articles subsequently screened for title and abstract, 95 were excluded based on having nonrelevant topics, employing nonrelevant tools, or being non-research articles. Finally, of the remaining 45 eligible studies, 32 were excluded for reasons including the absence of 2×2 tables, no index or reference test, and insufficient data. Thus, a total of 13 articles were identified in the database search and included in the meta-analysis. Also, manual searches via Google Scholar (*n* = 1) and citation searching (*n* = 12) yielded additional results, culminating in a total of 26 studies (Appendix F and Appendix G, Supplemental Digital Content, http://links.lww.com/JNR/A7). However, as three of these 26 articles included dual datasets, the total number of datasets used in this meta-analysis was 30.

The main characteristics of the included studies are summarized in Appendix F, Supplemental Digital Content, http://links.lww.com/JNR/A7. These 26 studies originated from nine different countries (Australia, Brazil, Canada, China, Finland, Portugal, Spain, Sri Lanka, and the United Kingdom) and were published between 2006 and 2021. The total number of participants across studies was 5,396, with 2,173 females and an average age of 60.2 years. The index tests used in these studies included NRS-2002 (*n* = 17), MUST (*n* = 4), and MST (*n* = 6). The reference standard, PG-SGA, was categorized into three ranges using the following cutoff points: PG-SGA ≥4, PG-SGA ≥9, and PG-SGA (B+C).

### Quality of Included Studies and Publication Bias

QUADAS-2 was employed in this meta-analysis to assess the quality of the included studies. Overall, no high concern was indicated regarding the risk of bias and applicability. In terms of bias risk, 74.1% of the included studies had low concern for patient selection, 51.9% had low concern for the index test, 55.6% had unclear concern for the reference standard, and 55.6% had low concern for flow and timing. Moreover, in terms of applicability, 92.6% of the studies had low concern for patient selection, 88.9% had low concern for the index test, and 70.4% had unclear concern for the reference standard. Overall, the included studies exhibited a low risk of bias in applicability (Appendix H, Supplemental Digital Content, http://links.lww.com/JNR/A7). In terms of Deeks’ funnel plot results, MST, MST, and NRS-2002 were all free of publication bias (*p*>.10; Appendix I, Supplemental Digital Content, http://links.lww.com/JNR/A7).

### Pooled Diagnostic Accuracy of the MST, MUST, and NRS-2002

Pooled estimates for sensitivity, specificity, +/LR, −/LR, DOR, and AUC were provided. The pooled sensitivity values for MST, MUST, and NRS-2002 were .78 (95% CI [.62, .88], .74 (95% CI [.68, .80]), and .67 (95% CI [.61, .71]), respectively, and the pooled specificity values were .82 (95% CI [.73, .89]), .80 (95% CI [.64, .90]), and .88 (95% CI [.83, .92]), respectively.

NRS-2002 had the highest +/LR and −/LR values of 5.70 (95% CI [4.00, 8.20]) and 0.38 (95% CI [0.33, 0.43]), respectively. MUST and MST had +/LR values of 3.70 (95% CI [1.80, 7.50]) and 4.40 (95% CI [2.80, 6.70]) and −/LR values of 0.32 (95% CI [0.23, 0.46]) and 0.27 (95% CI [0.15, 0.47]), respectively.

**Figure 2:**
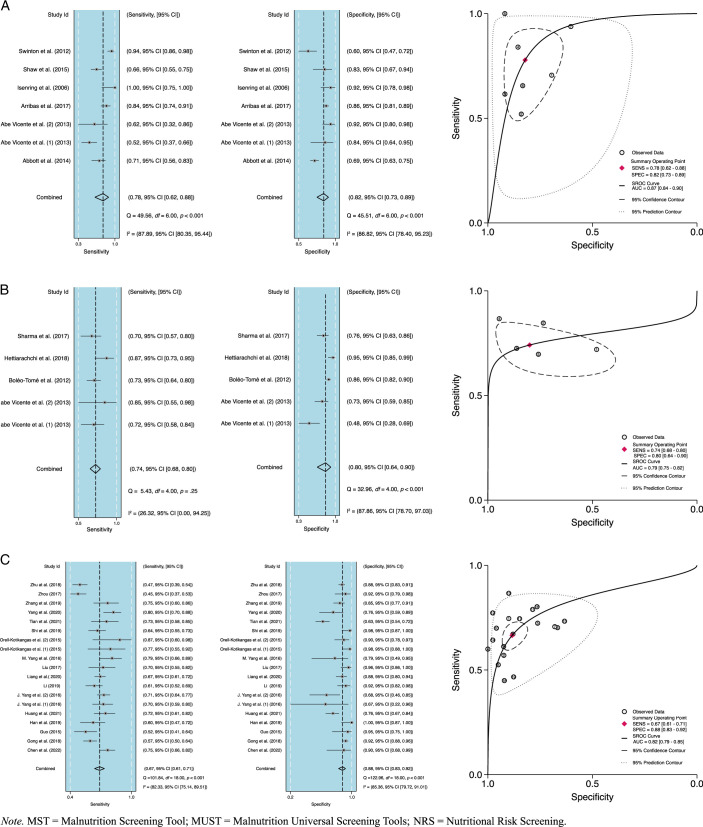
Forrest Plot and Summary Receiver Operating Characteristic Curves for MST (A), MUST (B), NRS-2002 (C)

The pooled DOR values for MST, MUST, and NRS-2002 were 16.00 (95% CI [7.00, 36.00]), 11.00 (95% CI [4.00, 32.00]), and 15.00 (95% CI [10.00, 23.00]), respectively. The pooled AUC values for MST, MUST, and NRS-2002 were .87 (95% CI [.84, .90]), .79 (95% CI [.75, .82]), and .82 (95% CI [.79, .85]), respectively. The summary receiver operating characteristic curve shown in Figure 2 illustrates the diagnostic efficiency, while the forest plots show the sensitivity and specificity of MST, MUST, and NRS-2002.

### Sensitivity Analysis

In this meta-analysis, a sensitivity analysis was conducted to assess the impact of measurement differences (Appendix J, Supplemental Digital Content, http://links.lww.com/JNR/A7). The goodness-of-fit test and bivariate normality analysis for all three screening tools support the robustness of the bivariate model and the appropriateness of its weighting. In addition, no outliers were detected. Both MST and MUST exhibited favorable analysis engagement values. However, the analysis of Zhu et al. (2018) on NRS-2002 generated data that did not align with the rest of the data used in this study.

### Results of Bayes Analysis

Fagan’s plots were used in this study to assess the post-test probability among malnourished patients with the pretest probability of 40% (Barker et al., 2011; Appendix K, Supplemental Digital Content, http://links.lww.com/JNR/A7). Based on Fagan’s plot results, for MST, a positive test result raises the probability of malnutrition to 74%, while a negative test result decreases it to 15%. For MUST, a positive test result increases the probability of malnutrition to 71%, while a negative test result decreases it to 18%. For NRS-2002, a positive test result elevates the probability of malnutrition to 79%, while a negative test result decreases it to 20%. Furthermore, the analysis of these three screening tools indicates their +LR is <10, and −LR is >0.1, implying all three screening tools have a minimal effect rating in detecting malnutrition among patients with chronic diseases.

## Discussion

This meta-analysis provides new information regarding the performance of MST, MUST, and NRS-2002, all of which are widely used in clinical settings to evaluate the nutritional status of inpatients with chronic diseases. MST was found to have good sensitivity and an AUC value higher than the other two screening tools. However, in terms of specificity, NRS-2002 earned a higher value than the other two. The findings in this study are credible and may be used by nurses, dietitians, and physicians to evaluate nutritional status in patients with chronic diseases.

The healing process of patients with chronic diseases is often disrupted due to the inaccurate interpretation of their nutritional status, which is an important component in the healing process. Many interpretation errors are due to the misinterpretation of health workers, such as nurses, dietitians, and physicians and inadequately sensitive instruments. Sensitivity in the context of malnutrition refers to the ability of a screening tool to identify individuals who are malnourished correctly. In this study, MST was shown to have relatively good sensitivity (.78, 95% CI [.62, .88]) and AUC (.87, 95% CI [.84, .90]) values compared with the other two instruments. In the context of malnutrition, specificity refers to the ability of a screening tool to correctly identify malnourished individuals. In this meta-analysis, all three screening tools were found to provide similar levels of specificity. MST plays a crucial role in the early detection of malnutrition and the timely implementation of interventions, significantly improving patient outcomes. MST’s objectivity and use of objective, standardized questions ensure consistent results across health care settings and practices, reliably identifying individuals at risk of malnutrition regardless of care setting. Developed to evaluate nutritional status in hospitalized, outpatient, and institutionalized adult patients, MST’s simplicity and small number of questions covering appetite, nutritional intake, and recent weight loss make it efficient to use (Ferguson et al., 1999). Furthermore, MST is a cost-effective instrument that is relatively simple to administer and does not require extensive resources or laboratory tests. Prior research has also affirmed MST as a validated screening tool with a high level of accuracy that is widely used in hospital admissions (Nakyeyune et al., 2021). Similarly, MUST was shown to exhibit good sensitivity (.74, 95% CI [.68, .80]) and AUC (.79, 95% CI [.75, .82]) values and to employ a minimal number of questions (Elia, 2003). However, MUST involves calculating BMI and weight loss, which requires specialized expertise, to complete, potentially explaining its lower sensitivity and specificity compared with MST (Totland et al., 2022). In addition, similar to the findings of a previous study, NRS-2002 earned the lowest sensitivity value (.67, 95% CI [.61, .71]) of the three (Wong et al., 2023). NRS-2002 used the most questions, required BMI calculations, and asked for the patient’s specific disease type (Kondrup et al., 2003), necessitating specific knowledge and skills to avoid errors during completion.

In this study, MST, MUST, and NRS-2002 were found to have significantly smaller effect ratings for risk of malnutrition detection in patients with chronic diseases than the reference standard (PG-SGA). This may be attributed to the limitations in the list of questions and standardized criteria used in these screening tools, which may not adequately encompass the diverse conditions of individual patients, thereby affecting instrument effectiveness. Beyond the questions posed, another factor contributing to the reduced effectiveness of these tools may be the number of patients and assessors involved (Totland et al., 2022). In addition, the limited sample sizes of the included studies may have been a factor underlying the lower effectiveness observed. To enhance the detection of malnutrition risk in patients with chronic diseases, health care providers may benefit from considering more comprehensive assessments that encompass disease-specific nutritional evaluations, detailed dietary histories, and collaboration with registered dietitians or nutrition specialists who possess expertise in managing malnutrition within the context of chronic illnesses. The development and utilization of diagnostic tools tailored to specific chronic diseases may also improve the accuracy of malnutrition risk assessments done on this patient population.

### Strengths and Limitations

This meta-analysis study was conducted to compare the performance of three screening tools widely used in clinical settings to assess the nutritional status of patients with chronic diseases. The strength of this research lies in its inclusion of studies from a cross-section of countries, which enhances the external validity of the analysis. Furthermore, the use of robust research methods helps ensure the reliability of the results obtained. However, one weakness of this study is that it encompasses several studies affected by poor information clarity, which may impact overall analysis results. Also, the small number of studies included for MST and MUST may raise concerns for further analysis. In addition, no moderator analysis was done in this study, which, if performed, may have identified covariate relationships affecting the performance of these three screening tools.

### Conclusions

The results of this study suggest that MST, MUST, and NRS-2002 all provide good accuracy in assessing the risk of malnutrition in patients with chronic diseases, with MST exhibiting a higher AUC value than the others. MST is a screening tool with a minimal number of questions, making it quick and straightforward to complete. However, the results of the Bayes analysis indicate MST, MUST, and NRS-2002 are associated with a lower effect rating than PG-SGA (the reference standard) in detecting risk of malnutrition in patients with chronic diseases. While sensitivity and specificity are crucial in understanding the diagnostic accuracy of a test, they must be interpreted within a broader context that includes other relevant metrics and considerations. Thus, filling these tools out accurately is crucial to preventing errors when interpreting/applying the results. In clinical settings, nurses may employ this instrument and collaborate with dietitians and other health care workers for support and assessment. Furthermore, given feasibility considerations, MST may be used together with the other two instruments for routine nutritional screening in daily practice. In future studies, covariates and additional evaluations should be explored to enhance internal validity.

## Supplementary Material
