# Supplementary material for: Performance of Malnutrition Screening Tools on People With Chronic Diseases: A Bivariate Meta-Analysis
Source: J Nurs Res. 2026 Feb 4;34(1):e440. doi: 10.1097/jnr.0000000000000728 (PMC12863617; doi:10.1097/jnr.0000000000000728)

**Appendix A**

**Examples of Search Terms Used in Embase and PubMed**

| Database | Population | Index Test | Reference Test | Outcome |
| --- | --- | --- | --- | --- |
| Embase | malnutrition  ‘nutritional deficiency’  ‘nutritional disorder’ | ‘mini-nutritional assessment short-form’  ‘nutritional risk screening 2002’  nrs-2002  ‘malnutrition universal screening tools’  must  ‘malnutrition screening tool’  mst | ‘patient generated subjective global assessment’  pg-sga | ‘diagnostic test’  ‘diagnostic accuracy’  diagnosis  ‘receiver operating characteristic’  sensitivity  speciﬁcity  validity  reliability  validation  ‘cut off value’  threshold  accuracy  psychometry |
| PubMed | Malnutrition  “Nutritional Deficienc*”  Undernutrition  Malnourishment*  “Nutritional Disease*”  “Nutrition Disorder*”  “Deficiency Disease*” | “Mini Nutritional Assessment Short Form”  MNA-SF  “Nutritional Risk Screening 2002”  NRS-2002  “Malnutrition Universal Screening Tools”  MUST  “Malnutrition Screening Tool”  MST | “Patient-Generated Subjective Global Assessment”  PG-SGA | “Diagnostic Tests, Routine”  diagnosis  “ROC Curve”  “Sensitivity and Specificity”  Validity  Reliability  “Data Accuracy”  Psychometrics |

**Appendix B**

**Examples of Syntax Used in Embase**

| Database | Syntax | Number | Total |
| --- | --- | --- | --- |
| Embase | #1: ‘malnutrition’/exp OR malnutrition OR ‘nutritional deficiency’:ti,ab,kw OR ‘nutritional disorder’:ti,ab,kw | 233,854 | 97 |
|  | #2: ‘mini-nutritional assessment short-form’/exp OR ‘mini-nutritional assessment short-form’ OR ‘nutritional risk screening 2002’:ti,ab,kw OR ‘nrs 2002’:ti,ab,kw OR ‘malnutrition universal screening tools’:ti,ab,kw OR must:ti,ab,kw OR ‘malnutrition screening tool’:ti,ab,kw OR mst:ti,ab,kw | 689,074 |  |
|  | #3: ‘patient generated subjective global assessment’/exp OR ‘patient generated subjective global assessment’ OR ‘pg sga’:ti,ab,kw | 1,187 |  |
|  | #4: ‘diagnostic test’/exp OR ‘diagnostic test’ OR ‘diagnostic accuracy’:ti,ab,kw OR diagnosis:ti,ab,kw OR ‘receiver operating characteristic’:ti,ab,kw OR sensitivity:ti,ab,kw OR speciﬁcity:ti,ab,kw OR validity:ti,ab,kw OR reliability:ti,ab,kw OR validation:ti,ab,kw OR ‘cut off value’:ti,ab,kw OR threshold:ti,ab,kw OR accuracy:ti,ab,kw OR psychometry:ti,ab,kw | 6,574,272 |  |
|  | #1 AND #2 AND #3 AND #4 | 97 |  |

**Appendix C**

*M***alnutrition Screening Tool – Questionnaire**

| Score | Question |
| --- | --- |
|  | Have you recently lost weight without trying? |
| 0 | No |
| 2 | Unsure |
|  | If yes, how much weight have you lost? |
| 1 | 2–13 lbs |
| 2 | 14–23 lbs |
| 3 | 24–33 lbs |
|  | Have you been eating poorly because of decreased appetite? |
| 0 | No |
| 1 | Yes |

*Note.* Score 0 or 1: not at malnutrition risk; if length of stay exceeds 7 days, re-screen and repeat as needed. Score of 2 or more: at malnutrition risk; rapidly implement nutrition interventions. Perform nutrition consult within 24–72 hours, depending on risk.

**Appendix D**

**Malnutrition Universal Screening Tools – Questionnaire**

| Point | Question |
| --- | --- |
|  | BMI ^a^ (Kg/m^2)^ |
| 0 | > 20 |
| 1 | 18.5–20 |
| 2 | < 18.5 |
|  | Unplanned weight loss in the past 3–6 months |
| 0 | < 5% |
| 1 | 5%–10% |
| 2 | > 10% |
|  | Acute disease effect ^b^ |
| 2 | A patient is acutely ill and there has been or is likely to be no nutrition intake for > 5days |

*Note.* To calculate the overall score, add all scores together and identify low risk (0 point), medium risk (1 point), and high risk (≥ 2 points). For complete detail questionnaire, please visit (<https://www.bapen.org.uk/pdfs/must/must_full.pdf>)

^a^ If unable to obtain height and weight, use alternative measurements and subjective criteria. ^b^ Acute disease effect is unlikely to apply outside the hospital.

**Appendix E**

**Nutritional Risk Screening 2002 – Questionnaire**

Initial Screening

| Indicator | Yes | No | |
| --- | --- | --- | --- |
| Is BMI < 20.5? |  | |  |
| Has the patient lost weight within the last 3 months? |  | |  |
| Has the patient had a reduced dietary intake in the last week? |  | |  |
| Is the patient severely ill? (e.g. in intensive therapy) |  | |  |

*Note.* Yes: If the answer is ‘Yes’ to any question, a final screening is performed; No: If the answer is ‘No’ to all questions, the patient is re-screened at weekly intervals. If the patient is scheduled for a major operation, a preventive nutritional care plan is considered to avoid associated risks.

Final Screening

| Point | Question |
| --- | --- |
|  | **Impaired nutritional status** |
| 0 | Normal nutritional status |
| 1 | Weight loss > 5% in 3 months or food intake below 50%–75% of normal requirements in preceding week |
| 2 | Weight loss > 5% in 2 weeks or BMI of 18.5–20.5 with impaired general condition or food intake 25%–50% of normal requirements in the preceding week |
| 3 | Weight loss > 5% in 1 month (> 15% in 3 months) or BMI < 18.5 with impaired general condition or food intake 0%–25% of normal requirements in preceding week |
|  | **Severity of disease (increase in requirements)** |
| 0 | Normal nutrition requirements |
| 1 | Hip fracture, chronic illness, or acute complications such as cirrhosis, COPD, chronic hemodialysis, diabetes, or cancer |
| 2 | Major abdominal surgery, stroke, severe pneumonia, or hematologic malignancy |
| 3 | Head injury, bone marrow transplant, and intensive care patients |

*Note.* The total score is calculated by adding the scores from impaired nutritional screening and disease severity. If the patient’s age is ≥ 70 years, add 1 to the total score.

Score ≥ 3: The patient is nutritionally at risk and a nutritional care plan is initiated.

Score < 3: Weekly rescreening of the patient. If the patient is scheduled for a major operation, a preventive nutritional care plan is considered to avoid associated risks.

BMI = body mass index; COPD = chronic obstructive pulmonary disease.

**Appendix F**

**Overall Characteristics of the Included Studies**

| No. | Study/Country | Tools | Comparator Tools | Settings | Diseases | Total Sample, Female (%), Age (mean), BMI (mean) |
| --- | --- | --- | --- | --- | --- | --- |
| 1 | Abbott et al. (2014)/  Australia | MST ≥ 2 | PG-SGA (B+C) | Hospital | Breast cancer, gastrointestinal cancer, hematological cancer, head and neck cancer, lung cancer, and others | Total sample: 300  Female: 48.3  Age: 58.6  BMI: 27.8 |
| 2 | Abe Vicente et al. (1) (2013)/  Brazil | MST ≥ 2 | PG-SGA (B+C) | Hospital | Colorectal cancer and gastric cancer | Total sample: 75  Female: 52.0  Age: 60.2  BMI: N/A |
| 3 | Abe Vicente et al. (2) (2013)/  Brazil | MST ≥ 2 | PG-SGA (B+C) | Hospital | Colorectal cancer and gastric cancer | Total sample: 62  Female: 54.8  Age: 61.3  BMI: N/A |
| 4 | Arribas et al. (2017)/  Spain | MST ≥ 2 | PG-SGA (B+C) | Hospital | Solid tumor and hematologic malignancies | Total sample: 394  Female: 44.9  Age: 61.6  BMI: N/A |
| 5 | Isenring et al. (2006)/  Australia | MST ≥ 2 | PG-SGA (B+C) | Hospital | Cancer | Total sample: 50  Female: 64.0  Age: 59.1  BMI: 26.5 |
| 6 | Shaw et al. (2015)/  United Kingdom | MST ≥ 2 | PG-SGA (B+C) | Cancer Centre | Haemato-oncology, gastrointestinal, breast, urology, gynecology, sarcoma, head and neck, lung, and other | Total sample: 126  Female: 54.0  Age: 59.0  BMI: 26.1 |
| 7 | Swinton et al. (2012)/  Canada | MST ≥ 2 | PG-SGA ≥ 4 | Oncology Clinic | Cancer | Total sample: 144  Female: 50.0  Age: 67.0  BMI: N/A |
| 8 | Abe Vicente et al. (1) (2013)/  Brazil | MUST ≥ 2 | PG-SGA (B+C) | Hospital | Colorectal cancer and gastric cancer | Total sample: 75  Female: 52.0  Age: 60.2  BMI: N/A |
| 9 | Abe Vicente et al. (2) (2013)/  Brazil | MUST ≥ 2 | PG-SGA (B+C) | Hospital | Colorectal cancer and gastric cancer | Total sample: 62  Female: 54.8  Age:61.3  BMI: N/A |
| 10 | Boléo-Tomé et al. (2012)/  Portugal | MUST ≥ 2 | PG-SGA (B+C) | Hospital | Cancer | Total sample: 450  Female: 40.0  Age: 62.0  BMI: N/A |
| 11 | Hettiarachchi et al. (2018)/  Sri Lanka | MUST ≥ 2 | PG-SGA (B+C) | Oncology Clinic | Breast cancer, ovary cancer, prostate cancer, lung cancer, thyroid cancer, gastrointestinal cancer, and others | Total sample: 100  Female: 68.0  Age: 58.6  BMI: 22.2 |
| 12 | Sharma et al. (2017)/  Australia | MUST ≥ 2 | PG-SGA (B+C) | Hospital | Respiratory diseases, cardiac problems, falls, CNS diseases, Other | Total sample: 132  Female: 62.9  Age: 79.5  BMI: N/A |
| 13 | Chen et al. (2022)/  China | NRS-2002 ≥ 3 | PG-SGA ≥ 9 | Hospital | Cancer | Total sample: 146  Female: 42.5  Age: 60.3  BMI: 21.7 |
| 14 | Gong et al. (2018)/  China ^a^ | NRS-2002 ≥ 3 | PG-SGA ≥ 4 | Hospital | Gastrointestinal tumors, head and neck tumors, others | Total sample: 423  Female: 37.4  Age: 56.2  BMI: N/A |
| 15 | Guo et al. (2015)/  China ^a^ | NRS-2002 ≥ 3 | PG-SGA ≥ 9 | Hospital | Colorectal cancer | Total sample: 100  Female: 43.0  Age: N/A  BMI: N/A |
| 16 | Han et al. (2019)/  China ^a^ | NRS-2002 ≥ 3 | PG-SGA (B+C) | Hospital | Gastric cancer | Total sample: 91  Female: 23.1  Age: N/A  BMI: N/A |
| 17 | Huang et al. (2021)/  China | NRS-2002 ≥ 3 | PG-SGA ≥ 9 | Hospital | Gastric cancer | Total sample: 181  Female: 36.5  Age: 55.3  BMI: N/A |
| 18 | J. Yang et al. (2016)/  China | NRS-2002 ≥ 3 | PG-SGA ≥ 4 | Hospital | Cancer | Total sample: 482  Female: 57.1  Age: 57.2  BMI: N/A |
| 19 | Li (2019)/  China ^a^ | NRS-2002 ≥ 3 | PG-SGA ≥ 4 | Hospital | Gastric cancer | Total sample: 187  Female: 37.4  Age: 57.0  BMI: N/A |
| 20 | Liang et al. (2020)/  China ^a^ | NRS-2002 ≥ 3 | PG-SGA ≥ 4 | Hospital | Colorectal cancer | Total sample: 392  Female: 36.7  Age: 62.6  BMI: N/A |
| 21 | Liu (2017)/  China ^a^ | NRS-2002 ≥ 3 | PG-SGA (B+C) | Hospital | Esophageal cancer, gastric cancer, colon cancer, pancreatic cancer, gallbladder cancer | Total sample: 99  Female: 47.5  Age: 62.3  BMI: N/A |
| 22 | M. Yang et al. (2016)/  China ^c^ | NRS-2002 ≥ 3 | PG-SGA (B+C) | Hospital | Gastric cancer | Total sample: 71  Female: 26.8  Age: 58.1  BMI: N/A |
| 23 | Orell-Kotikangas et al. (1) (2015)/  Finland | NRS-2002 ≥ 3 | PG-SGA (B+C) | Hospital | Head and neck squamous cell carcinoma | Total sample: 65  Female: 23.0  Age: N/A  BMI: N/A |
| 24 | Orell-Kotikangas et al. (2) (2015)/  Finland | NRS-2002 ≥ 3 | PG-SGA ≥ 9 | Hospital | Head and neck squamous cell carcinoma | Total sample: 65  Female: 23.0  Age: N/A  BMI: N/A |
| 25 | Shi et al. (2019)/  China ^a^ | NRS-2002 ≥ 3 | PG-SGA ≥ 4 | Hospital | Gastric cancer | Total sample: 168  Female: 22.6  Age: N/A  BMI: 23.1 |
| 26 | Tian et al. (2021)/  China | NRS-2002 ≥ 3 | PG-SGA ≥ 4 | Hospital | Cancer | Total sample: 165  Female: N/A  Age: N/A  BMI: N/A |
| 27 | Yang et al. (2020)/  China ^a^ | NRS-2002 ≥ 3 | PG-SGA ≥ 4 | Hospital | Lung cancer | Total sample: 120  Female: N/A  Age: 63.9  BMI: N/A |
| 28 | Zhang et al. (2019)/  China ^a^ | NRS-2002 ≥ 3 | PG-SGA ≥ 9 | Hospital | Head and neck tumors, chest tumors, digestive system tumors, gynecological tumors | Total sample: 163  Female: 34.4  Age: 53.2  BMI: N/A |
| 29 | Zhou (2017)/  China ^a^ | NRS-2002 ≥ 3 | PG-SGA (B+C) | Hospital | Gastric Cancer | Total sample: 196  Female: 29.6  Age: 59.3  BMI: N/A |
| 30 | Zhu et al. (2018)/  China ^a^ | NRS-2002 ≥ 4 | PG-SGA ≥ 4 | Hospital | Breast cancer, malignant lymphoma, head and neck cancer, and other | Total sample: 466  Female: 55.2  Age: 50.6  BMI: 22.2 |

*Note.* Detailed references for the included studies are given in the appendix G.

MST = Malnutrition Screening Tools, MUST = Malnutrition Universal Screening Tools, NRS-2002 = Nutritional Risk Screening 2002, PG-SGA = Patient-Generated Subjective Global Assessment, BMI = Body Mass Index, N/A = Data not available.

^a^ The values for True Positive, True Negative, False Positive, and False Negative were retrieved from (Ruan et al., 2021)

.

**Appendix G**

**Reference List of Included Studies**

Abbott, J., Teleni, L., McKavanagh, D., Watson, J., McCarthy, A., & Isenring, E. (2014). A novel, automated nutrition screening system as a predictor of nutritional risk in an oncology day treatment unit (ODTU). *Supportive Care in Cancer*, *22*(8), 2107-2112. <https://doi.org/10.1007/s00520-014-2210-7>

Abe Vicente, M., Barão, K., Silva, T. D., & Forones, N. M. (2013). What are the most effective methods for assessment of nutritional status in outpatients with gastric and colorectal cancer? *Nutricion Hospitalaria*, *28*(3), 585-591. <https://doi.org/10.3305/nh.2013.28.3.6413>

Arribas, L., Hurtós, L., Sendrós, M. J., Peiró, I., Salleras, N., Fort, E., & Sánchez-Migallón, J. M. (2017). NUTRISCORE: A new nutritional screening tool for oncological outpatients. *Nutrition*, *33*, 297-303. <https://doi.org/10.1016/j.nut.2016.07.015>

Boléo-Tomé, C., Monteiro-Grillo, I., Camilo, M., & Ravasco, P. (2012). Validation of the Malnutrition Universal Screening Tool (MUST) in cancer. *British Journal of Nutrition*, *108*(2), 343-348. <https://doi.org/10.1017/S000711451100571X>

Chen, Y., Xiang, Q., Li, C., Zeng, Y., Dong, J., Zhang, P., Li, Y., Wang, Y., & Wang, K. (2022). Nutritional Risk and Assessment for Patients with Cancer Pain. *Nutrition & Cancer*, *74*(1), 168-174. <https://doi.org/10.1080/01635581.2021.1882510>

Gong, L.-Q., Liu, N., Wang, Y.-L., Xin, X.-W., Zong, X.-L., Sun, Y., Jiang, L.-L., Zhang, X.-T., Yue, H.-Z., & Fang, Y. (2018). Nutritional assessment in oncology patients: A comparative analysis between NRS 2002 and PG-SGA. *Electronic Journal of Metabolism and Nutrition of Cancer*, *5*(2), 151-154. <https://doi.org/10.16689/j.cnki.cn11-9349/r.2018.02.008>

Guo, T. (2015). *100 cases of colorectal cancer patients nutrition status quo investigation and study.* Hebei Medical University.

Han, F., Bian, X., Chen, D., Ge, W., & Gua, W. (2019). Comparison of applicability for different nutritional screening methods in gastric cancer patients undergoing gastrectomy. *Pharmaceutical and Clinical Research*, *27*(6), 449-453. <https://doi.org/10.13664/j.cnki.pcr.2019.06.012>

Hettiarachchi, J., Madubhashini, P., & Miller, M. (2018). Agreement between the Malnutrition Universal Screening Tool and the Patient-Generated Subjective Global Assessment for cancer outpatients receiving chemotherapy: A cross-sectional study. *Nutrition & Cancer*, *70*(8), 1275-1282. <https://doi.org/10.1080/01635581.2018.1539186>

Huang, D. L., Wu, X. H., Wang, C. L., Chen, J. Q., Jia, K., Zhou, J., Lu, L. S., Zhang, Y. S., Jin, J., & Huang, K. K. (2021). Relationship of the preoperative NRS 2002 score, PG-SGA score, and serum indices with postoperative complications in patients with gastric cancer. *Journal of Nutritional Oncology*, *6*(2), 74-80. https://doi.org/[10.34175/jno202102004](https://doi.org/10.34175/jno202102004)

Isenring, E., Cross, G., Daniels, L., Kellett, E., & Koczwara, B. (2006). Validity of the malnutrition screening tool as an effective predictor of nutritional risk in oncology outpatients receiving chemotherapy. *Supportive Care in Cancer*, *14*(11), 1152-1156. <https://doi.org/10.1007/s00520-006-0070-5>

Li, G. (2019). *The correlation study of nutritional related blood biochemical indexes and anthropometry indexes in nutrition screening and evaluation in patients with gastric cancer* [Unpublished master's thesis]. Guangxi Medical University, China.

Liang, H., Wu, W., Jia, S.-Y., Zeng, C., Cheng, G., Jiang, X.-D., Shen, J., Xu, B.-F., & Qin, K. (2020). Investigation on nutritional status of patients with colorectal cancer and nutritional intervention of pharmacists. *Practical Pharmacy and Clinical Remedies*, *23*(7), 632-636. <https://doi.org/10.14053/j.cnki.ppcr.202007012>

Liu, Y. (2017). *Application's comparison of NRS-2002 and PG-SGA in the ﬁrst chemotherapy of patients with advanced digestive system neoplasms.* Shanxi Medical University.

Orell-Kotikangas, H., Österlund, P., Saarilahti, K., Ravasco, P., Schwab, U., & Mäkitie, A. A. (2015). NRS-2002 for pre-treatment nutritional risk screening and nutritional status assessment in head and neck cancer patients. *Supportive Care in Cancer*, *23*(6), 1495-1502. <https://doi.org/10.1007/s00520-014-2500-0>

Sharma, Y., Thompson, C., Kaambwa, B., Shahi, R., & Miller, M. (2017). Validity of the Malnutrition Universal Screening Tool (MUST) in Australian hospitalized acutely unwell elderly patients. *Asia Pacific Journal of Clinical Nutrition*, *26*(6), 994-1000. <https://doi.org/10.6133/apjcn.022017.15>

Shaw, C., Fleuret, C., Pickard, J. M., Mohammed, K., Black, G., & Wedlake, L. (2015). Comparison of a novel, simple nutrition screening tool for adult oncology inpatients and the Malnutrition Screening Tool (MST) against the Patient-Generated Subjective Global Assessment (PG-SGA). *Supportive Care in Cancer*, *23*(1), 47-54. <https://doi.org/10.1007/s00520-014-2319-8>

Shi, Y.-Y., & Bian, X.-J. (2019). Comparison of preoperative nutritional screening and evaluation by NRS2002 and PG-SGA in gastric cancer patients. *Practical Journal of Medicine & Pharmacy*, *36*, 501-503. <https://doi.org/10.14172/j.issn1671-4008.2019.06.007>

Swinton, N., Kasymjanova, G., Grossman, M., Cohen, V., Pepe, C., Agulnik, J. S., & Small, D. (2012). Validation of the patient-generated part of the PG-SGA against the Malnutrition Screening Tool (MST). *Supportive Care in Cancer*, *20*, S264. <https://doi.org/10.1007/s00520-012-1479-7>

Tian, M., Fu, H., & Du, J. (2021). Application value of NRS2002 and PG-SGA in nutritional assessment for patients with cervical cancer surgery. *American Journal of Translational Research*, *13*(6), 7186-7192.

Yang, H., Han, X., Ran, G., & Jin, X. (2020). Application of NRS2002 and PG-SGA in lung cancer patients undergoing non-surgical treatment. *Chongqing Medical Journal*, *14*(10), 1677-1680. <https://doi.org/10.3969/j.issn.1671-8348.2020.10.030>

Yang, J., Yuan, K., Huang, Y., Yu, M., Huang, X., Chen, C., Fu, J., Shi, Y., & Shi, H. (2016). Comparison of NRS 2002 and PG-SGA for the assessment of nutritional status in cancer patients. *Biomedical Research (India)*, *27*(4), 1178-1182.

Yang, M., Chen, Y., Wang, X.-J., Chen, J.-H., & Guo, Z.-Q. (2016). The application of NRS 2002 and PG-SGA in chemotherapy patients with advanced gastric cancer. *Electronic Journal of Metabolism and Nutrition of Cancer*, *3*(1), 45-48.

Zhang, Y., Wang, Y., He, X., Wang, Z., Wang, N., & Fang, Y. (2019). Investigation on nutritional status of cancer patients during radiotherapy. *Chinese General Practice Nursing*, *17*(25), 3181-3184. <https://doi.org/10.12104/j.issn.1674-4784.2019.25.036>

Zhou, Y. (2017). *Follow-up study on nutritional status of patients with gastric cancer on different stages of treatment.* Southeast University.

Zhu, C., Wang, B., Gao, Y., & Ma, X. (2018). Prevalence and relationship of malnutrition and distress in patients with Cancer using questionnaires. *BMC Cancer*, *18*(1), Article No. 1272. <https://doi.org/10.1186/s12885-018-5176-x>

**Appendix H**

**Quality Assessment of Included Studies Using Quality Assessment Tool for Diagnostic Accuracy Studies-2**

| Study | Risk of Bias | | | | Applicability Judgment | | | |  |
| --- | --- | --- | --- | --- | --- | --- | --- | --- | --- |
|  | Patient Selection | Index Test | Reference Standard | Flow and Timing | Patient Selection | Index Test | | Reference Standard |  |
| MST | | | | | | | | |  |
| Abbott et al. | L | L | L | L | L | L | | L |  |
| Abe Vicente et al. | U | U | L | L | L | L | | L |  |
| Arribas et al. | L | L | L | L | L | L | | L |  |
| Isenring et al. | L | L | L | L | L | L | | L |  |
| Shaw et al. | L | L | L | L | L | L | | U |  |
| Swinton et al. | L | U | L | U | L | L | | L |  |
| MUST | | | | | | | | |  |
| Abe Vicente et al. | U | U | U | L | L | L | | L |  |
| Boléo-Tomé et al. | L | U | U | U | L | L | | L |  |
| Hettiarachchi et al. | L | U | U | U | L | L | | L |  |
| Sharma et al. | L | U | L | L | L | U | | L |  |
| NRS-2002 | | | | | | | | |  |
| Chen et al. | L | U | U | L | L | | L | L | |
| Gong et al. | L | L | L | U | L | | L | L | |
| Guo | L | L | U | U | L | | L | U | |
| Han et al. | L | L | U | U | L | | L | U | |
| Huang et al. | L | U | U | L | L | | L | L | |
| J. Yang et al. | U | U | U | L | L | | L | L | |
| Li | L | L | L | U | L | | L | L | |
| Liang et al. | L | L | U | U | L | | L | U | |
| Liu | U | L | L | L | U | | L | L | |
| M. Yang et al. | L | L | U | U | L | | L | U | |
| Orell-Kotikangas et al. | L | L | L | L | L | | L | L | |
| Shi et al. | L | L | U | U | L | | U | U | |
| Tian et al. | U | U | U | L | L | | L | L | |
| Yang et al. | L | U | U | U | L | | U | U | |
| Zhang et al. | L | L | U | U | L | | L | U | |
| Zhou | U | U | U | L | U | | L | L | |
| Zhu at al. | U | U | L | L | L | | L | L | |

*Note.* MST = Malnutrition Screening Tools, MUST = Malnutrition Universal Screening Tools, NRS-2002 = Nutritional Risk Screening 2002; L = low; U = unclear

**Appendix I**

**Deeks’ Funnel Plots of (A) MST, (B) MUST, and (C) NRS-2002**

*Note.* MST = Malnutrition Screening Tools, MUST = Malnutrition Universal Screening Tools, NRS-2002 = Nutritional Risk Screening 2002.

**
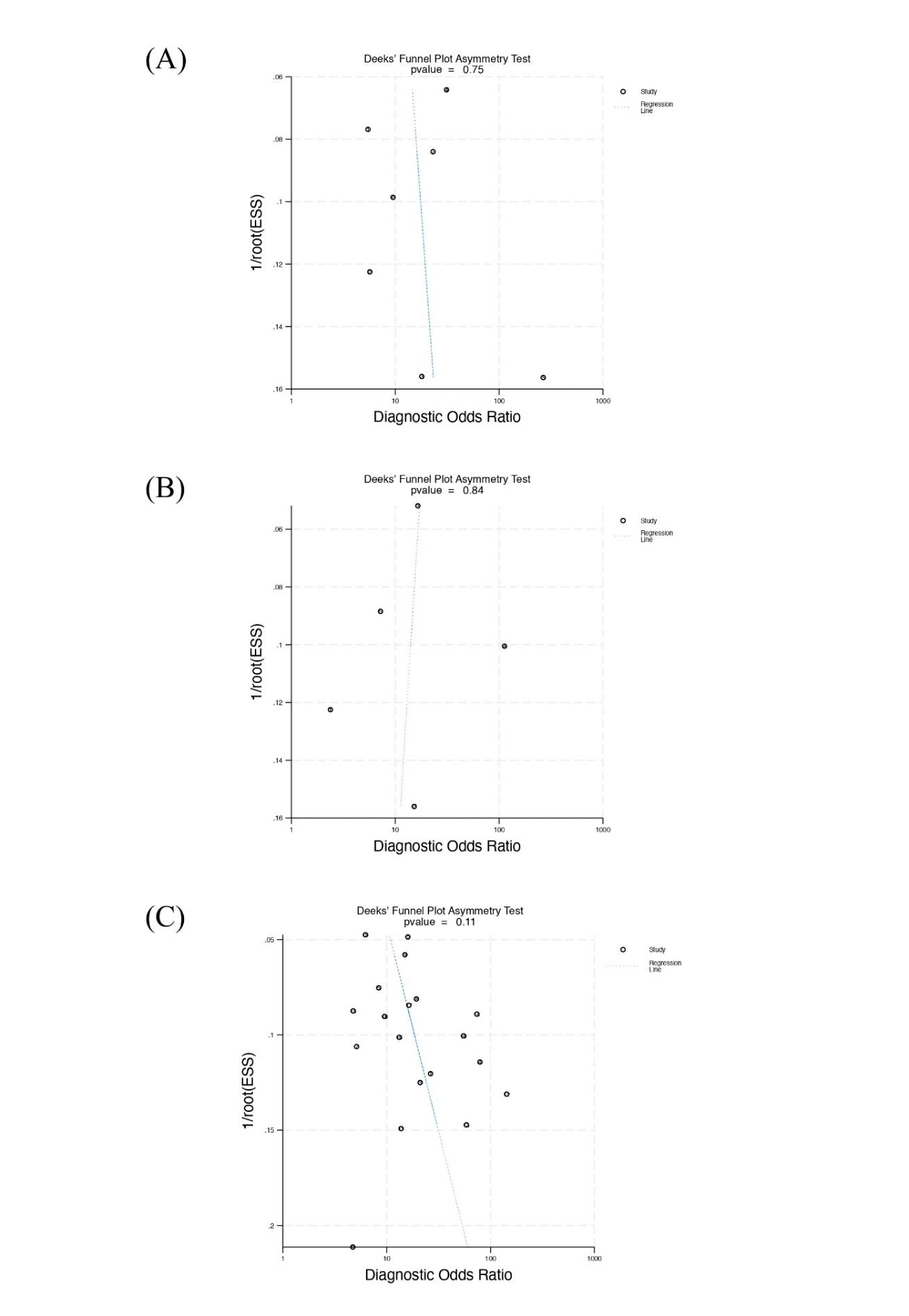
**

**Appendix J**

**Sensitivity Analysis of (A) MST, (B) MUST, and (C) NRS-2002**


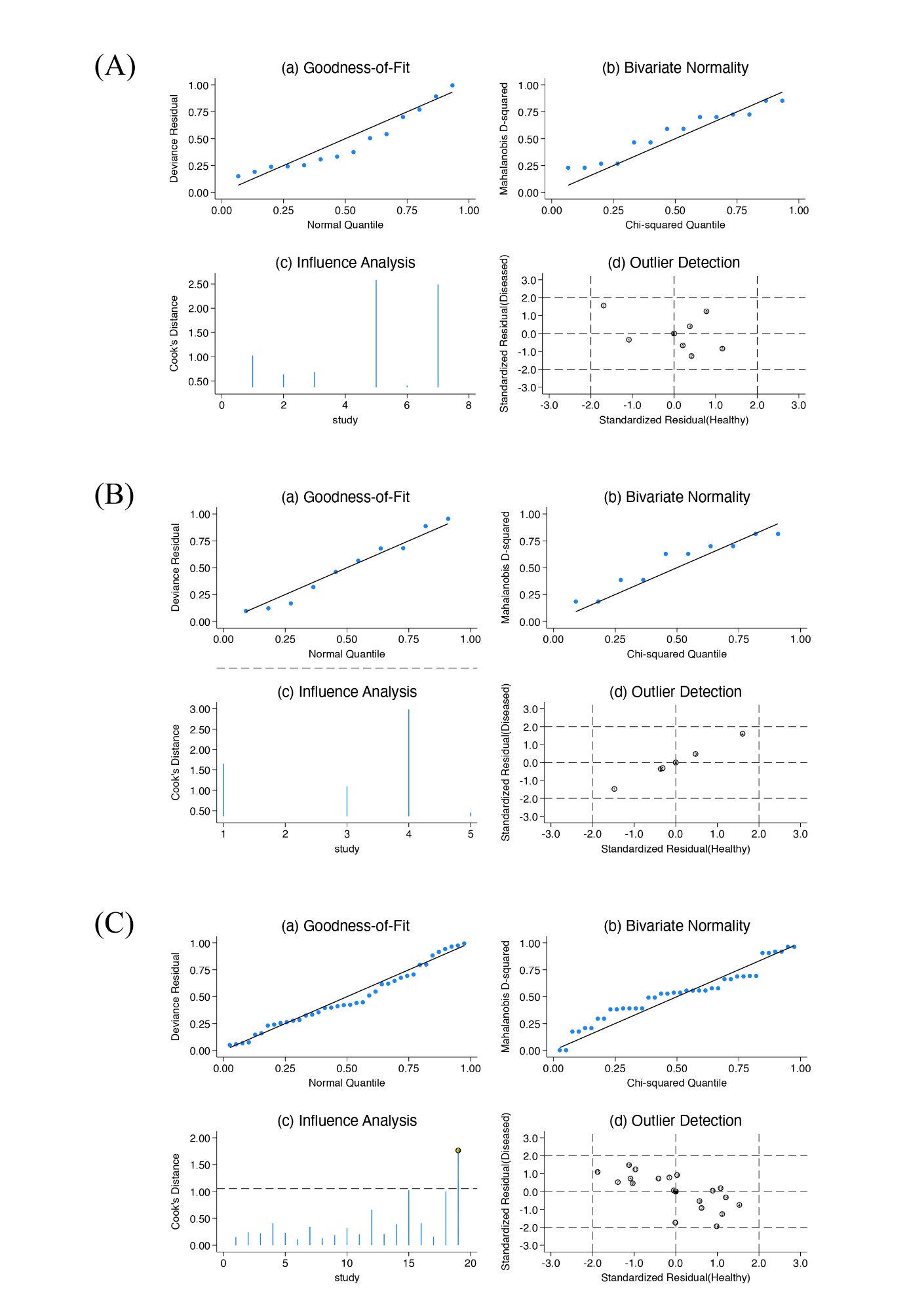


*Note.* MST = Malnutrition Screening Tools, MUST = Malnutrition Universal Screening Tools, NRS-2002 = Nutritional Risk Screening 2002.

**Appendix K**

**Fagan’s Plots to Evaluate the Diagnosis Accuracy of Malnutrition Screening Tools for Patients With Chronic Diseases Using (A) MST, (B) MUST, (C) NRS-2002. The Vertical Axes (1) on the Left Represents the Pre-Test of Probability, (2) in the middle Represents the Likelihood Ratio, and (3) on the Right Represents the Post-Test of Probability**


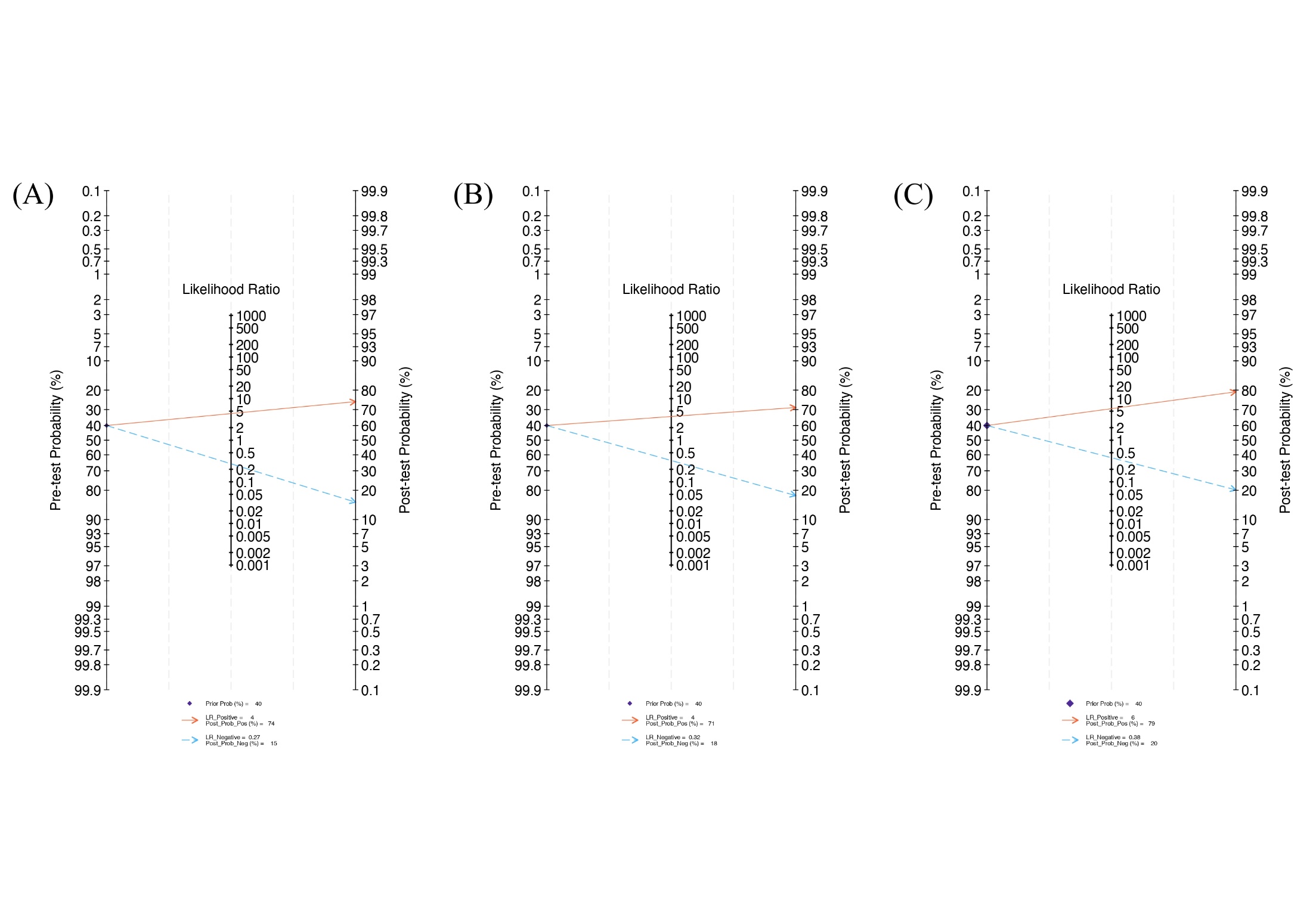

Supplement: Supplementary file 1 [file jnr-34-e440-s001.docx]
